# Data-driven mechanistic framework with stratified immunity and effective transmissibility for COVID-19 scenario projections

**DOI:** 10.1016/j.epidem.2024.100761

**Published:** 2024-03-21

**Authors:** Przemyslaw Porebski, Srinivasan Venkatramanan, Aniruddha Adiga, Brian Klahn, Benjamin Hurt, Mandy L. Wilson, Jiangzhuo Chen, Anil Vullikanti, Madhav Marathe, Bryan Lewis

**Affiliations:** a Biocomplexity Institute & Initiative, University of Virginia, Charlottesville, 22911, VA, USA; b Department of Computer Science, University of Virginia, Charlottesville, 22904, VA, USA

**Keywords:** COVID-19, Scenario projections, Compartmental model, Transmission dynamics, Variant characteristics, Population immunity

## Abstract

Scenario-based modeling frameworks have been widely used to support policy-making at state and federal levels in the United States during the COVID-19 response. While custom-built models can be used to support one-off studies, sustained updates to projections under changing pandemic conditions requires a *robust*, *integrated*, and *adaptive* framework. In this paper, we describe one such framework, **UVA-adaptive**, that was built to support the CDC-aligned Scenario Modeling Hub (SMH) across multiple rounds, as well as weekly/biweekly projections to Virginia Department of Health (VDH) and US Department of Defense during the COVID-19 response. Building upon an existing metapopulation framework, PatchSim, **UVA-adaptive** uses a calibration mechanism relying on adjustable effective transmissibility as a basis for scenario definition while also incorporating real-time datasets on case incidence, seroprevalence, variant characteristics, and vaccine uptake. Through the pandemic, our framework evolved by incorporating available data sources and was extended to capture complexities of multiple strains and heterogeneous immunity of the population. Here we present the version of the model that was used for the recent projections for SMH and VDH, describe the calibration and projection framework, and demonstrate that the calibrated transmissibility correlates with the evolution of the pathogen as well as associated societal dynamics.

## 1. Introduction

Mathematical models are increasingly being used by policymakers to help plan for and respond to public health outbreaks (Lofgren et al., 2014; Biggerstaff et al., 2022; Adiga et al., 2020). Such computational models have proven useful for outbreak responses at various spatial and jurisdictional scales. These models can be used to highlight key biological factors (Saad-Roy et al., 2020), predict future outcomes (Nixon et al., 2022), and estimate the effectiveness of available intervention strategies (Rivers et al., 2014). However, there are many unknowns during an infectious disease outbreak which pose modeling challenges, such as the biology of the pathogen (Wikramaratna et al., 2015; Lloyd-Smith et al., 2015) or the behavior of the population (Funk et al., 2015). These need to be integrated into the model and continuously updated to support long-term efforts, which is further complicated by the changing data and policy landscapes, that require the modeling framework to be flexible and capable of rapid updates.

Epidemic modeling strategies can be broadly classified into two main categories: mechanistic models and data-driven/statistical approaches (Adiga et al., 2020). Structured mechanistic models have advantages over purely data-driven methods, such as enhanced explainability, the ability to explore counterfactual scenarios, and the representation of specific behaviors. While mechanistic models excel at capturing essential epidemic mechanisms and projecting future dynamics, data-driven approaches often lack explicit interpretability, prioritizing their predictive capacity instead. However, in a prolonged pandemic, such as COVID-19, and the unprecedented level of gathered data in recent times, data-driven approaches are particularly empowered to provide useful information in the face of uncertainty within large interactive evolving systems (e.g. behavior, travel, social, and policy changes). Due to the complementary nature of these approaches, powerful synergies can be realized. For example, statistical models can be used to dynamically tune and refine mechanistic components, both over time and as additional data sources become available. Data-driven approaches can provide a formal means to quickly compare “what if” situations applied to efficient and robust mechanistic model components.

**Fig. 1.**
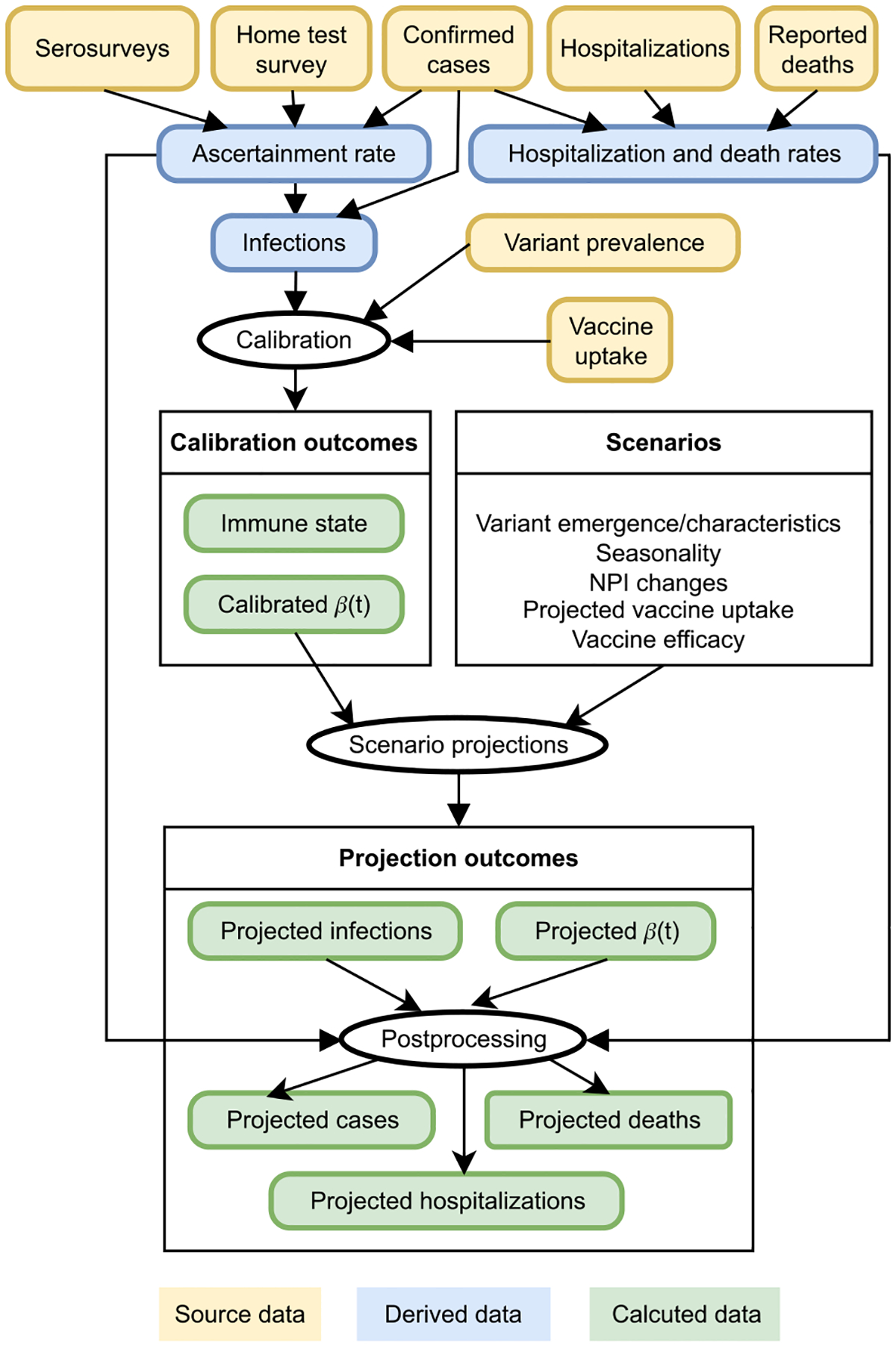
The overview of the data processing workflow for the UVA-adaptive.

To facilitate the scenario-based COVID-19 projections for Virginia Department of Health (VDH), Department of Defense, and the COVID-19 Scenario Modeling Hub (SMH), we have created a data-driven, mechanistic model named **UVA-adaptive**. This new model is based on our PatchSim metapopulation SEIR model (Venkatramanan et al., 2019) and is expanded to handle multiple strains and complex immunity. To calibrate the model and generate scenario-based projections we have added adaptive trajectory fitting and developed data-driven processing pipeline to accommodate evolution of the epidemic dynamics (Fig. 1). This approach incorporates evolving data sources with the metapopulation model and allows real-time, daily updates at a high resolution (up to county level). Here we present the core model that was used for the SMH rounds 11–16. We describe the calibration procedure and outcome generation for SMH and analyze the ability of this process to capture changing transmission dynamics. We also show how these kinds of models can estimate the immunity profile of the population and illustrate the model’s concordance with external data sources not included in the fitting process. We conclude by discussing the applicability of these kinds of models for scenario-based projections.

## 2. Materials and methods

The simulation model used for COVID-19 Scenario Modeling Hub (SMH) projections (COVID-19 Scenario Modeling Hub, 2021–2022; Borchering et al., 2021; Truelove et al., 2021) has evolved over time in response to data availability and scenario specification. It started as a discrete time *S**usceptible-**E**xposed-**I**nfectious-**R**ecovered* (SEIR) compartmental model with modeled Vaccination. In contrast to our earlier version that incorporated inter-county travel and mixing (Venkatramanan et al., 2019), we modeled each region (US state) independently and used an effective transmissibility β(t) to capture time-varying social distancing, variable prevalence/incidence, and policy and mobility effects across regions.

To accommodate evolving scenarios, we extended that version first by including multiple vaccination tiers and partial susceptibilities to capture vaccination and infection history, then by incorporating the layers corresponding to different strains’ infections. In the following sections we present this extended disease model and describe the parameters, calibration procedure and the processing that was used for the SMH projections in rounds 11–16.

### 2.1. Disease model

**Fig. 2.**
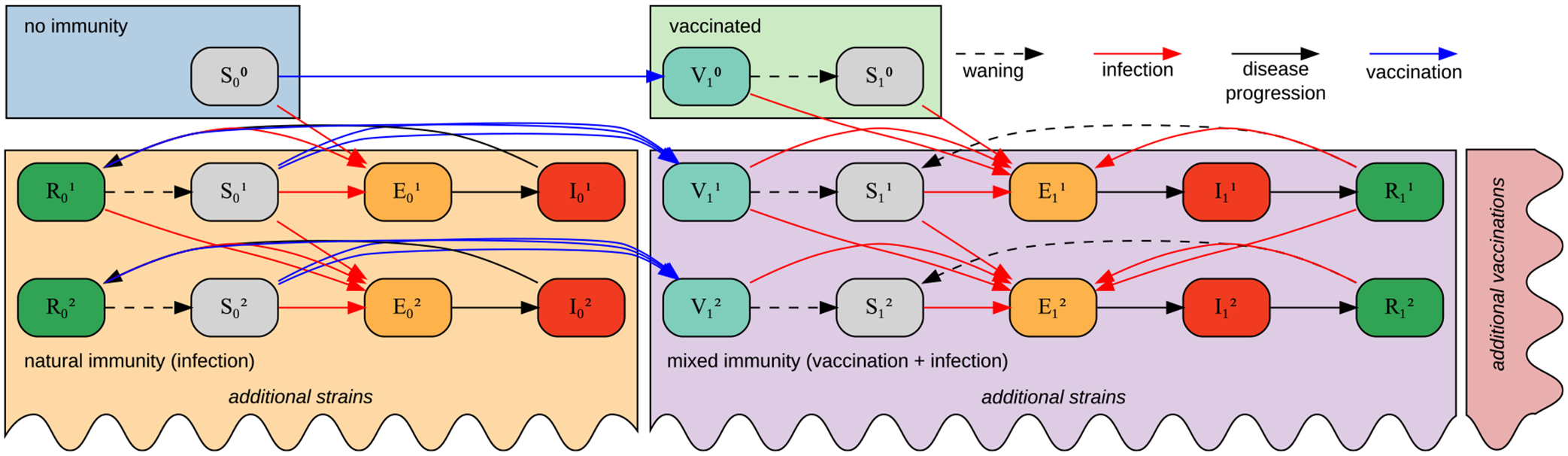
Example state transition graph of a two-strain, single vaccination event m∈{0,1,2},i∈{0,1}, model. The model can be extended by arbitrary number of vaccination events and variants.

The disease model can be described as ${\left(\text{SEIRV}\right)}_{i}^{m}$, where i represents vaccination status (number of doses) and m infecting strain. For a strain m∈M, and current, next and previous vaccination doses i,j,k we begin with the slightly modified SEIR dynamic to account for vaccination, waning of immunity and partial protection/immune escape. (See Fig. 2 for an example transition graph and supplementary materials for the expanded equations for the two strain, single vaccination model).

(1) $${\dot{S}}_{i}^{m}\;=-x\left(t,S_{i}^{m}\right)+\mu_{r}R_{i}^{m}+\mu_{v}V_{i}^{m}-v_{j}\left(t,S_{i}^{m}\right)$$

(2) $${\dot{E}}_{i}^{m}\;=x^{m}\left(t,S_{i},R_{i},V_{i}\right)-\alpha E_{i}^{m}$$

(3) $${\dot{I}}_{i}^{m}=\alpha E_{i}^{m}-\gamma I_{i}^{m}$$

(4) $${\dot{R}}_{i}^{m}\;=\gamma I_{i}^{m}-\mu_{r}R_{i}^{m}-x\left(t,R_{i}^{m}\right)-v_{j}\left(t,R_{i}^{m}\right)$$

(5) $${\dot{V}}_{i}^{m}\;=v_{i}\left(t,S_{k}^{m},R_{k}^{m},V_{k}^{m}\right)-\mu_{v}V_{i}^{m}-x\left(t,V_{i}^{m}\right)-v_{j}\left(t,V_{i}^{m}\right)$$

where $x^{m}\left(t,\cdot \right)$ represent the number of new exposures to strain m from all vaccination tiers, with $x\left(t,\cdot \right)=\sum_{n\in S}x^{n}\left(t,\cdot \right)$ exposures to all strains from a given source population (S,R, or V states). $v_{i}\left(t,\cdot \right)$ is the number of vaccinations to a target population (S,R, or V states) obtained by proportionately dividing the total vaccinations of dose i at time $t,v_{i}\left(t\right)$ across all eligible populations. The i=0 dose represents unvaccinated state. $\frac{1}{\alpha }$ and $\frac{1}{\gamma }$ are the mean durations of incubation and infectiousness respectively, and $\left\{\mu_{r},\mu_{v}\right\}$ represent the rates of waning from natural infection and vaccination. We use a “leaky” model of vaccine efficacy and partial protection after infection, therefore:

(6) $$x^{m}\left(t,S_{i}^{n}\right)=\rho_{mni}^{S}\beta \left(t\right)S_{i}^{n}I^{m}$$

(7) $$x^{m}\left(t,R_{i}^{n}\right)=\rho_{mni}^{R}\beta \left(t\right)R_{i}^{n}I^{m}$$

(8) $$x^{m}\left(t,V_{i}^{n}\right)=\rho_{mni}^{V}\beta \left(t\right)V_{i}^{n}I^{m}$$

where β(t) represents the transmissibility varying over time, which incorporates the effect of ongoing interventions and behavioral adaptations, as well as changing biological characteristics of the pathogen (e.g., increased transmissibility of new variants); $\rho_{mni}^{S},\rho_{mni}^{R}$, and $\rho_{mni}^{V}$ are partial susceptibilities against strain m after waning, after recovering from infection and partial susceptibility due to vaccine effectiveness, respectively, adjusted for the immune profile of the given state (strain of prior infection n and vaccination dose i). The partial susceptibilities after infection depend on cross-immunity between different strains. Based on observed lack of re-emergence of earlier strains, we have assumed sequential progression of the strains, i.e. infection with the new strain confers the highest immunity against infection with the historically preceding strains. For example, infection with BA.2 confers the same immunity against BA.1 as infection with the BA.1. This simplifies the model and ensures that the preceding strains do not reemerge in subsequent waves.

The structure of the above model does not explicitly capture presymptomatic/asymptomatic infections; however, we model these through a time-varying case-ascertainment rate (modeled infection to confirmed case ratio) and fitted β(t) to implicitly model asymptomatic infections and changes in infectiousness, respectively.

The direct outcomes generated by the above model are incident exposures x(t).

### 2.2. SMH projections

The projection targets of the SMH rounds 11–16 included cases, hospitalizations and deaths. To model these outcomes we have employed a calibration procedure that modified the β(t) at the state level, run the core model simulation and transformed the resulting calculated incident exposures x(t) into simulated reported cases. The resulting calibrated β(t) time series was used for scenario projections to generate case projection targets, which were further transformed to hospitalizations and deaths targets. The exact type of the model (number of strains and vaccination events) depend on the SMH round and is summarized in Table 1, and the values of the model parameters and datasources are provided in supplementary Tables S1 and S2.

#### Calibration.

The calibration procedure involves modifying the weekly transmissibility β(t) to minimize the absolute error between the simulated c(t) and reported cases C(t).

(9) $$\underset{\beta (t)}{\text{min}}\left|c\left(t+\delta_{c}\right)-C\left(t+\delta_{c}\right)\right|$$

(10) $$c\left(t+\delta_{c}\right)=x\left(t\right)\eta^{\prime }\left(t+\delta_{c}\right)$$

where $\eta^{\prime }$ is home-test adjusted case-ascertainment rate (see below) and $\delta_{c}$ is a time from infection to confirmation.

The above procedure is repeated for each region, to update the transmissibility matrix β(t) to current time points in a sequential fitting procedure. For each t, the estimation is done using the Golden Section Search (SciPy v1.8.0 Manual, 2022). If the $\left|c\left(t+\delta_{d}\right)-C\left(t+\delta_{d}\right)\right|<\epsilon$ (ϵ=1 case/day / 100k people) we keep the current β(t), otherwise we run GSS to optimize it. For the next period, we start from β(t)=β(t−1). The model is deterministic in nature (does not have inherent stochasticity) and probabilistic projections are obtained through an experiment design capturing parametric uncertainty. We used factorial design to capture uncertainty and variability in the following parameters: ascertainment rate η} (region dependent, +/−20%), time from infection to confirmation ($\delta_{c}\in \left[4,12\right]$ days), mean duration of incubation ($\frac{1}{\alpha }\in \left[5,9\right]$ days, [3.5, 6.3] days starting with Omicron), and duration of infectiousness ($\frac{1}{\gamma }\in \left[2,7\right]$ days, [1.4, 4.9] days starting with Omicron) resulting in 420 independent simulations with different parameter sets (cells). Cells that lead to poor fitting due to unrealistic parameters are filtered out, to create an ensemble of fitted β(t) across cells. For SMH reporting purposes, we calculate percentiles across cells for each t separately.

#### Case-ascertainment rate.

To derive region-specific time-varying case-ascertainment rates, we use nationally administered seroprevalence surveys (CDC, 2022). These surveys estimate the percentage of people p(t) in the United States with past SARS-CoV-2 infections during the testing period. Based on two testing timepoints $t_{0}$ and $t_{1}$, the case-ascertainment rate for the region during $\left[t_{0},t_{1}\right]$ period can be estimated as:

(11) $$\eta \left(t_{1}\right)=\frac{C\times \left(t_{1}\right)-C\left(t_{0}\right)}{P\times \left(p\left(t_{1}\right)-p\left(t_{0}\right)\right)}$$

where the C(t) are the cumulative confirmed cases for the region on time t, and P is the region total population. Due to potential antibody waning, testing sensitivity over time and changes in administered tests, we calculate variable rates until Jan 2021, then we assume that the rate is constant until wider availability of the home tests. Starting in October 2021, we adjust the case-ascertainment rate, using the home test use survey (Rader et al., 2022), as follows:

(12) $$\eta^{\prime }\left(t\right)=\eta \left(t\right)\left(1+\frac{N_{ht}/N_{0}\cdot f_{ht}}{f_{pcr}}\right)$$

where $N_{ht}/N_{0}$ is the ratio of tested people who self-reported using home tests to those who did not and $f_{ht,pcr}$ is the test positivity rate for antigen home test (self-reported) and PCR tests (reported by CDC).

#### Variant seeding, prevalence and immunoscape parameters.

To seed and maintain the variant prevalence, we use the prevalence data from CDC, using the following variants and variant groupings (pre-Omicron variants, BA.1, BA.2, BA.2.12.1, {BA.4/5, BA.2.75}, {BQ.1, BA.2.75.2}, BQ.1.1). For the purpose of SMH rounds, we use forced prevalence, i.e. at each iteration t, all new exposures x(t) are redistributed among all m strains so $x^{m}\left(t\right)$ matches the actual or modeled prevalence. Before round 16, we use modeled prevalence, which is expressed as a logistic function $q\left(t\right)=\frac{1}{1+e^{-k_{q}\left(t-t_{q}\right)}}$, where $k_{q}$ is the growth rate of the logistic function, and $t_{q}$ is the midpoint, where $k_{q}$ and $t_{q}$ are adjusted to fit observed data or set to scenario specifications (COVID-19 Scenario Modeling Hub, 2021–2022). For round 16, we use actual prevalence data published by CDC. For the initial period, we maintain steady seeding of 1 infection per day per 1M people. If the scenario specifies the seeding of a new variant, we distribute prescribed seeds among regions according to the population size. While in the earlier rounds we modulated the strain infectivity by modifying strain-inherent transmissibility, starting with round 11 we used the current model where we used immune escape only and allowed our calibrated β(t) to capture potential changes in strain transmissibility, unless the transmissibility changes were prescribed by the scenarios. For modeling purposes, we assumed that the highest immune escape is 80% and assigned that to all Omicron lineages vs. pre-Omicron. We used immune escape estimated from the published neutralization titers (Cao et al., 2022) (see Supplementary Material for explanation) As the study available at the time of estimation was limited to previous BA.1 infection, we used the relative reductions in $NT_{50}$ as an approximation, which likely overestimates immune escape.

#### Counterfactual projections.

One of the primary uses of a model structure like the one described above is the ability to capture the impact of various pharmaceutical/non-pharmaceutical interventions, behavioral/policy adaptations, and possible phase changes (e.g., novel variants) on the future time course of the epidemic. Similar frameworks have been used by multiple modeling groups (Fox et al., 2022; Reiner et al., 2021; Borchering et al., 2021) to do scenario-based modeling at various time points in the pandemic.

Counterfactual scenarios are simulated under different assumptions about β(t), variant prevalence and vaccination uptake/effectiveness, in the future. For instance, the *status quo* projections are obtained by using a smoothed version of recent β(t) into the future. The projected β's for each cell in the experiment design are further randomized with 5 samples taken from the uniform distribution [0.85β,1.15β], resulting in 2100 individual projection trajectories.

**Fig. 3.**
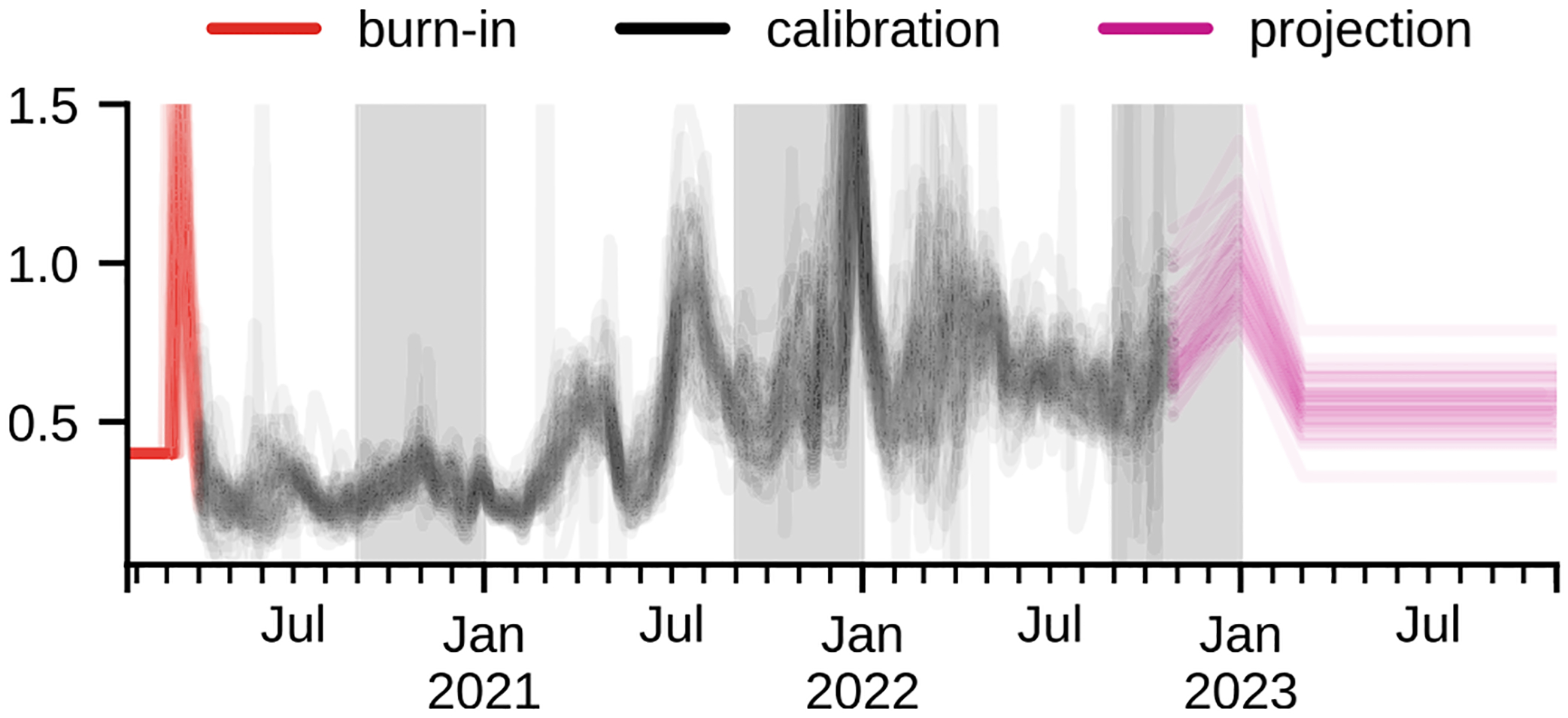
The median β(t) for all US states. Out of 2100 particles the median β(t) was calculated per week per region. Each line corresponds to a single US state. The periods assumed to correspond to seasonal increase are marked with gray background.

We assumed that changes in fitted β(t) for the period 2020-09-01 – 2021-03-01 correspond primarily to seasonal fall/winter effects (both environmental and behavioral) and use this assumption to model future seasonal changes. Based on that, we approximated the fall/winter change as a linear increase of β(t) starting from 2022-09-01 (or the last observed data point, whichever was later in the season) to the 1.6x baseline levels (defined as lower quartile of β(t) observed or projected in 2022-08) at 2022-12-31, then linearly decreasing to the baseline at 2023-03-01 (Fig. 3).

For the effective vaccinations time series v(t), we project vaccine uptake $U_{v}^{m,d}\left(t\right)$ by region, manufacturer, and dose to asymptotically reach observed regional vaccine acceptance rates (Salomon et al., 2021) over the projection horizon using monotonic cubic interpolation (Fritsch and Butland, 1984). For other expert-informed scenarios (COVID-19 Scenario Modeling Hub, 2021–2022), we appropriately adjust the model parameters to capture future alterations to Non-Pharmaceutical Interventions (NPIs), vaccination rates and projected variants.

#### Incident hospitalization and death estimates.

Given the fitted model and scenario-based projections, we obtain corresponding hospitalization and death time series through post-processing. For each round casehospitalization-ratio (CHR, $\rho^{H}$) and case-fatality-ratio (CFR, $\rho^{D}$), as well as the time differences $\left(\delta_{h},\delta_{g}\right)$ between case and hospitalization/death reporting were simultaneously estimated for each region through a least-squares fitting procedure and recent data. In this simple form,

(13) $$h\left(t\right)=\rho^{H}c\left(t-\delta_{h}\right)$$

(14) $$d\left(t\right)=\rho^{D}c\left(t-\delta_{d}\right)$$

Next, the impact of a novel variants is incorporated by scaling $\rho^{H}$ and $\rho^{D}$ by its relative severity (for example, 0.7 for Omicron for round 12, pessimistic severity scenarios (COVID-19 Scenario Modeling Hub, 2021–2022)) and projected prevalence q(t). Finally, the modeled hospitalizations/deaths were scaled to match the last 2 weeks of reported data.

### 2.3. Additional analyses

#### Transmissibility analysis.

The median regional $\beta \left(t\right)\left(\tilde{\beta }\left(t\right)\right)$ was used to fit the apparent transmissibility advantage of SARS-CoV-2 variants circulating during the analysis period. We used variant proportions calculated from the GISAID data, as provided by outbreak.info, and grouped into major variants using the pango_aliasor package. These resulted in the following variants that had at least 10% prevalence in one of the US states: A/B, Alpha, Beta, Gamma, Delta, Epsilon, Iota, Mu, Omicron.

The prevalence of these strains was used to create a multivariate linear regression (MLR) model that expresses the $\tilde{\beta }\left(t\right)$ as a linear combination of variant prevalence. From the coefficients, we have calculated the apparent transmissibility increase.

#### Correlation analysis.

To check correlation of the fitted transmissibility with other, independently recorded signals, we used the variant-corrected transmissibility time series (residual from the MLR, $\hat{\epsilon }\left(t\right)$) and differenced $\hat{\epsilon }\left(t\right)$ time series. We used state-level Google Mobility Reports (Google LLC, 2022), mask-wearing trends from “The Delphi Group at Carnegie Mellon University U.S. COVID-19 Trends and Impact Survey, in partnership with Facebook” (Salomon et al., 2021), and temperature and humidity (state level median weekly values from weather stations in NOAA Integrated Surface Database (ISD), NOAA National Centers for Environmental Information (2023)). We also performed the correlation analysis with the differenced versions of the time series, since, for some signals, the changes could correlate well with a change in transmissibility. Single correlation coefficient was calculated for the analyzed time period (2020-04-03 – 2022-10-14 for mobility and weather signals and 2020-09-11 – 2022-07-01 for mask wearing). Weekly median signal values were used.

## 3. Results

The multi-strain, multi-vaccination core model was first used during COVID-19 SMH round 11 (first of the Omicron rounds). The data and the parameters continued to be modified for subsequent rounds. The same model structure, applied at the county and district levels, was used for the projections for Virginia Department of Health (Virginia Department of Health, 2022). The final outcomes (cases, hospitalizations, deaths) of the model were submitted for the SMH, are available at SMH website and are evaluated as part of the SMH wide effort (Howerton et al., 2023). In the following sections we focus our analyses on the fitted transmissibility and the underlying model states.

### 3.1. Fitted transmissibility

**Fig. 4.**
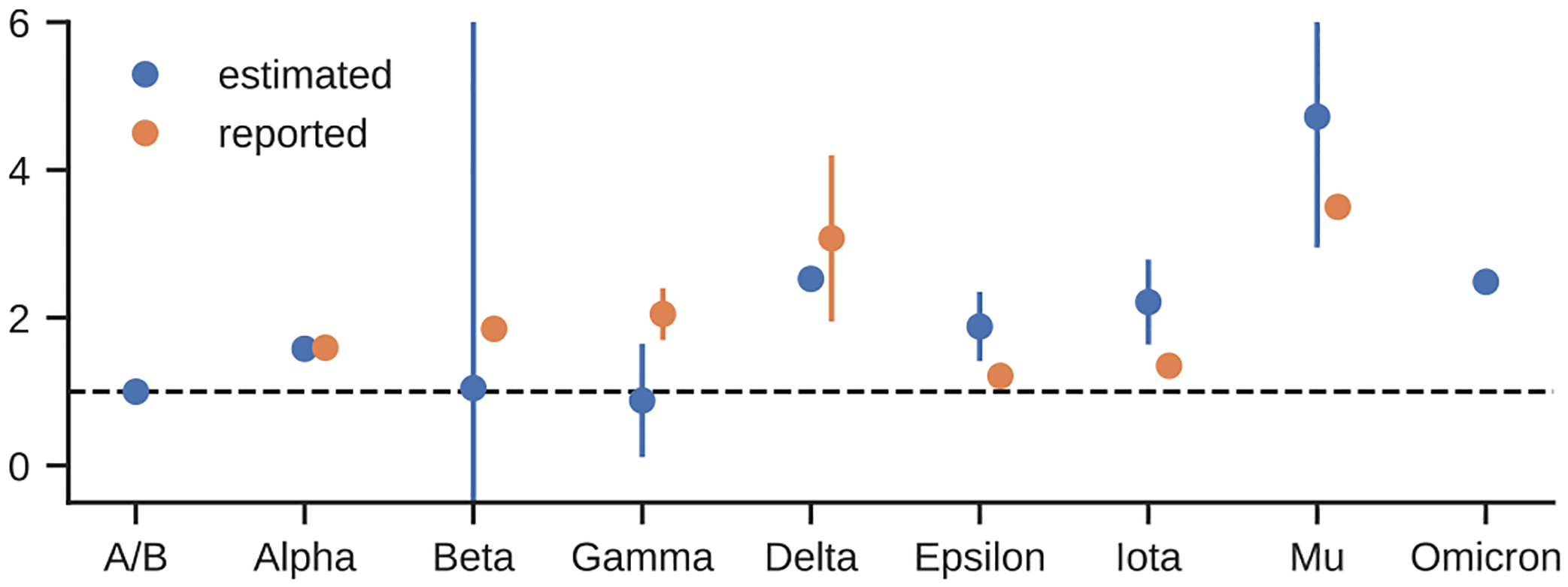
The apparent transmissibility increase of the analyzed SARS-CoV-2 variants estimated from the fitted β(t).

As discussed above, the fitted β(t) (Fig. 3) reflects a combination of factors, including strain transmissibility changes in the long term, effect of ongoing NPI, as well as short-term fluctuations due to behavioral changes. The fitted β(t) captures effects that are not included in the model and/or misrepresented in the used data. We intentionally did not model the strain transmissibility changes in the model; therefore, it is possible to validate the fitting procedure by extracting the transmissibility increase that can be attributed to pre-Omicron strains (see 2.3, Fig. 4). We observe that the fitted values for major variants circulating in the US (Alpha and Delta) agree with the transmissibility advantage reported elsewhere (Table 2. The remaining variants were present only in several states with low variant proportion. The fitted and reported values agree for the Mu variant and the Epsilon and Iota variants are close but overestimated.

### 3.2. Population immune profile

**Fig. 5.**
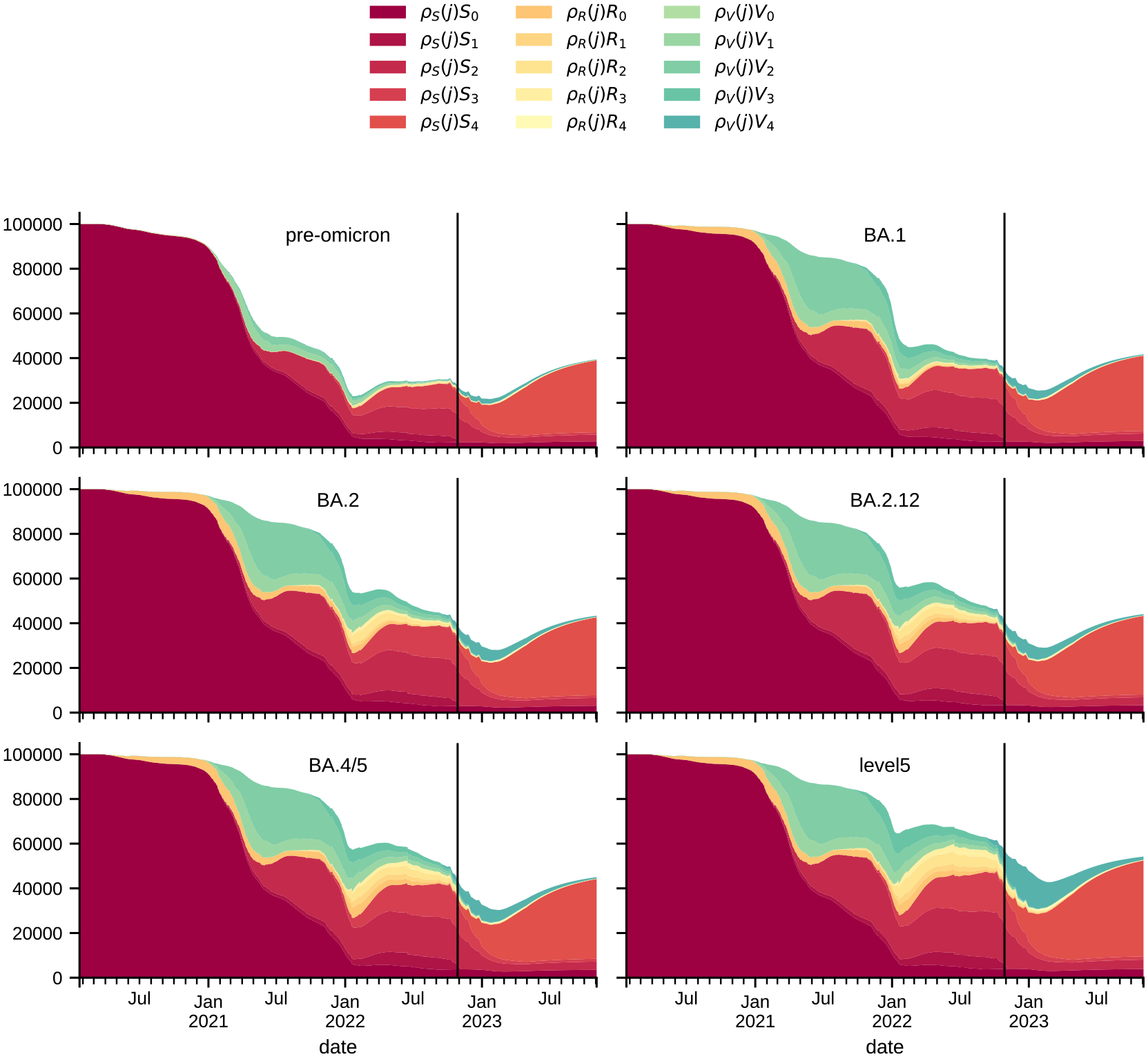
Effective susceptible population (per 100,000) for Virginia, for US wide plot see Figure S6. The vertical line marks the end of calibration period and start of the projection.

The underlying model structure allows for extraction of the population immunity (or susceptibility) profile divided into immunological tiers corresponding to vaccination tiers represented in the model and prior infection history (Fig. 5). The population susceptible to a particular variant comes from different compartments due to increasing immune escape, and depends on the time from the introduction of the previous variant. For example, introduction of the Omicron strain significantly increased effective susceptible populations coming from recently vaccinated states, while introduction of “Level 5” Omicron lineage (as defined during SMH Round 16) increased susceptibility in those previously infected as compared to the profile of population susceptibility to BA.4/5.

**Fig. 6.**
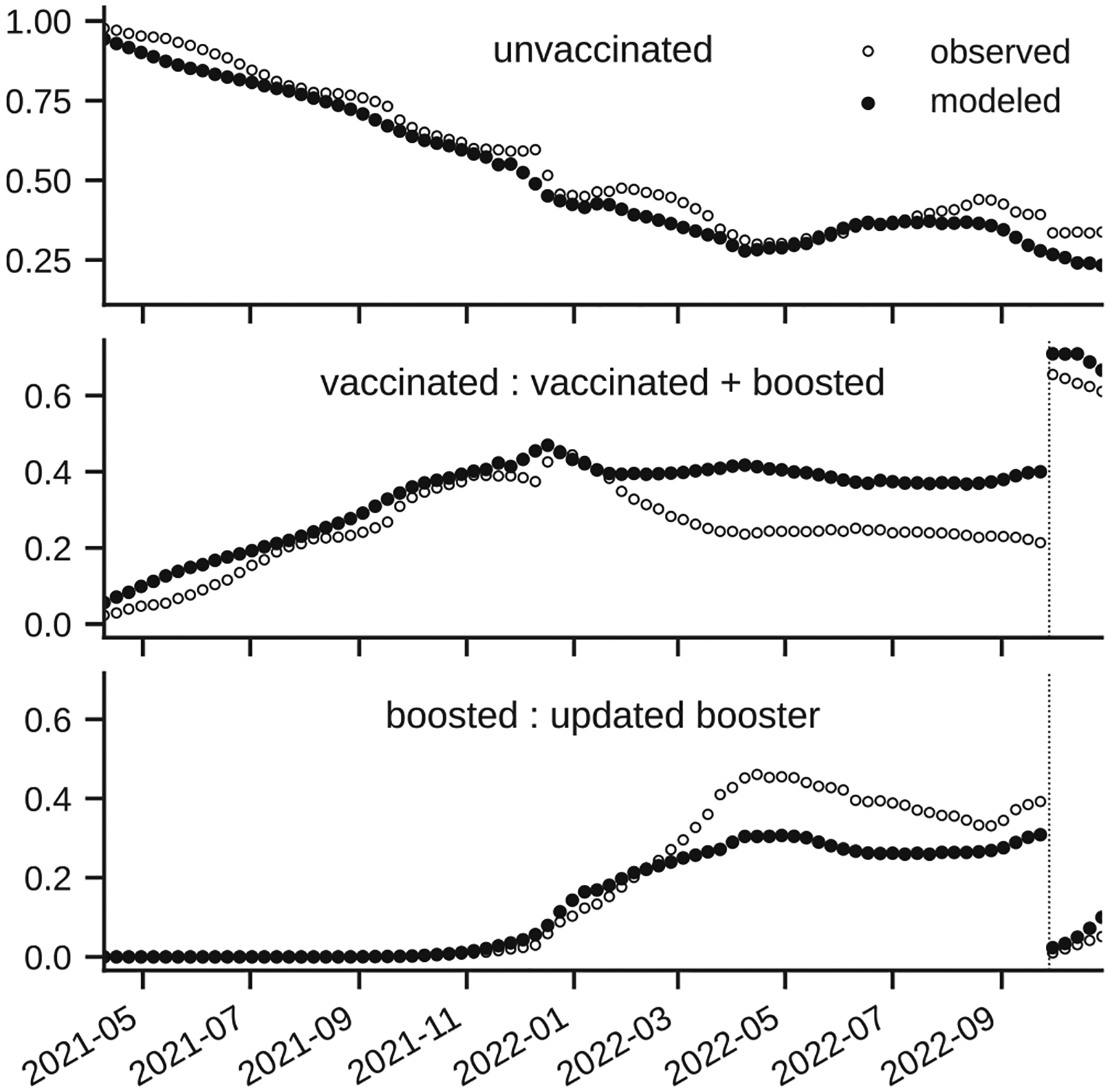
Fraction of infections coming from different vaccinated states compared to the fraction of reported cases reported by CDC. Note that, in Oct 2022, CDC included boosters in the vaccinated strata, this switch has been marked with the vertical dotted line.

This allows for the extraction of the infections coming from different vaccination states. Comparison with breakthrough case data published by the CDC shows that the proportion of modeled infections coming from the unvaccinated population follows the published data (Fig. 6). However, the proportion of infections coming from the population vaccinated with the primary series compared to the population who received boosters starts to diverge during the Omicron wave, and our model underestimates the number of infections coming from the boosted population. One of the possible explanations is that, for these scenarios, we kept the vaccine efficacy of both the primary series and the boosters against infection with the Omicron lineages constant (the scenario allowed flexibility in assumed immune escape for the recently boosted population). Furthermore, for this period, we applied ascertainment rate correction factors based on the home test surveys (informed by data from OutbreaksNearMe (Rader et al., 2022) since the seroprevalence data was discontinued in February 2022) uniformly across all regions.

### 3.3. Correlation with other signals

**Fig. 7.**
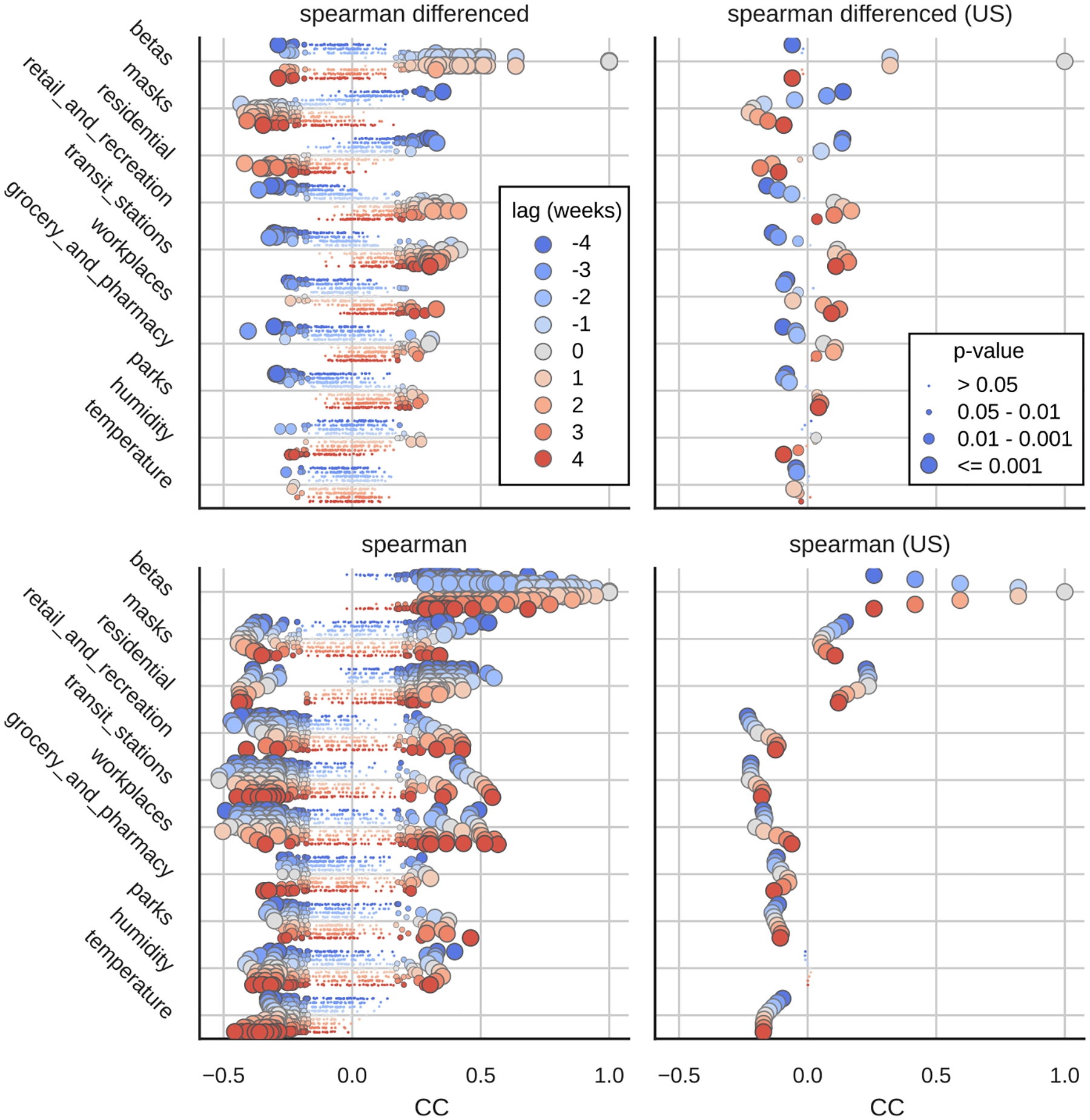
Spearman Correlation Coefficients between strain corrected β(t) (or δβ(t)) and different lags of selected signals for each US state (left side) or US wide (right side). The lag of 4 means that the signal was shifted 4 weeks forward relative to β(t)(β(t)~signal(t+4weeks)). For each signal and lag, single point represent single US state and the point size correspond to the P-value. For full matrix of correlation coefficients and their P-values see Figures S2 to S5.

We also studied the correlations of fitted β(t) corrected by the variant specific contributions ($\hat{\epsilon }\left(t\right)$, see 4, Figure S1) with other signals that capture epidemiological relevant aspects such as mobility, mask-wearing, and seasonality (Fig. 7). We have observed that at the US level (all weekly time points from all states analyzed together) all variables except non-differenced humidity had a significant (P-value < 0.05) Spearman correlation coefficient (SCC). The highest correlation between the differenced time series was for the mask usage levels, which had the best SCC (across states) for the one week lagged time series. The β(t) and mask usage had a negative correlation for positive lags, indicating that the decrease in the mask usage was associated with the increase in the β(t) after lag period. Changes in mask usage also had a high positive CC with β(t) when using negative delays, suggesting that the drop in transmissibility over time (highest correlation after 4 weeks) correlates with decreased mask usage. At the state level, all states except DC, Idaho, Kentucky, Maine, New York and Wyoming had a significant negative SCC with changes in mask usage at positive lags and some states had significant positive SCC with changes in mask usage at −3 and −4 lags, however, none of the states had a significant SCC with inverse relationship (positive SCC with positive lags and negative SCC with negative lags)

The differenced mobility signals (7, top panels) displayed lower SCC than mask usage. At the country level, the mobility signals (retail, transit, workplaces, grocery and parks) with negative lags had the negative SCC (highest after 3–4 weeks) and positive lags had the positive SCC (highest after 2–3 weeks), indicating that the decrease/increase in β(t) correlated after 3–4 weeks with increase/decrease in mobility and that increase/decrease in mobility correlated after 2–3 weeks with an increase/decrease in β(t). As expected, the reverse relationship was observed with mobility associated with residential areas (inoriginalcrease/decrease in β(t) was correlated with increase/decrease in residential mobility).

The weather signals correlated weakly with the betas regardless of applied delay, with non-differenced temperature (7, bottom panels), having the highest negative correlation with 4 week delay.

## 4. Discussion

We have developed UVA-adaptive a compartmental mechanistic model with multiple immune states that can represent a heterogeneous population with multiple vaccination events and multiple strains. This model has been used for regular projections for VDH and US COVID-19 SMH, its projections were used as input for Bayesian Ensemble Model forecasts submitted to COVID-19 Forecast Hub and served as a basis for calculating medical resource demand. **UVA-adaptive** is a realization of the hybrid mechanistic and data-driven approach, where the basic disease dynamics are modeled using an explainable mechanistic compartmental SEIR-like model, and the remaining factors are captured using a data-driven fitting procedure. This approach enabled us to provide operational projections that capture multiple factors, even as we were still expanding the underlying SEIR model. In this paper, we have presented the model structure, analyze its ability to capture factors not explicitly included in the calibration and modeling process such as complex immunity landscape, strain transmissibility changes, mobility changes and behavioral adaptations. In the following sections we discuss potential and limitations of the model and calibration procedure to capture these factors in the context of scenario-based projections.

### 4.1. Model

Due to the complexity of the spatiotemporal calibration process, the model was restricted to treating each local population independently and approximate all external influences by fitting the β(t). Due to lack of connectivity between the different regions, inter-regional transmissions (especially for novel emerging variants) needed to be implemented through external seeding. Another limitation of the current model is a lack of explicit age stratification. Similar to spatial effects, the lack of appropriate age-specific contact matrices prevents us from capturing the varying transmission dynamics due to different NPIs (e.g., school closures) that affected age groups in an asymmetric manner. Furthermore, age distribution in confirmed cases varied across the different waves, but real-time data on this across states was sparse. Instead of modeling each age group separately, we modeled aggregate population dynamics and included any age-related effects (for example, age-dependent hospitalization ratio) during post-processing. Finally, our model does not explicitly include any behavioral effects (such as NPI effects, adaptive behavior) and to be included in projections, such effects must be expressed as changes in β(t). If there is a corresponding period in the past that can be used to evaluate the effects, then the changes can be expressed as relative changes in the β(t) that are inferred from the past fitted β(t). If such a period is not available, the possible changes need to be evaluated externally, perhaps through a detailed agent-based model (see Chen et al. *in review*).

### 4.2. Data/calibration

The analysis of the fitting results demonstrates that our calibration procedure that modifies only single parameter β(t) can capture the changes related to the evolution of the COVID-19 variants and changes in the societal dynamics. The one “catch-all” parameter and the calibration procedure have proved to be robust from operational perspective and allowed us to provide constant updates with reasonable flexibility to implement various scenarios; however, the limited calibration procedure was heavily dependent on the external data sources, estimation of the other parameters, and inclusion of the parameters in the factorial design. For example, the multi-strain model for the SMH rounds depends on the immune escape matrix and strain-specific vaccine efficacies. They were estimated from early data and not calibrated and were not updated during the rounds. This is evident in the discrepancy between the fraction of infections coming from unvaccinated, vaccinated and boosted populations. Due to lack of detailed data at the design stage about the vaccine efficacy against different Omicron variants we have used the same protection levels for all Omicron variants and that likely resulted in the observed discrepancy between the proportion of infections coming from vaccinated wrt. boosted population that is highest for the BA.2 variant.

Finally, the uncertainty of the model is based on different trajectories resulting from factorial design but does not include additional uncertainty resulting from the parameters not included in the design (such as waning immunity). Due to the potential underestimation of uncertainty, the projections are obtained from trajectories using perturbed β(t).

The calibrated β(t) is able to capture the relative changes related to strain transmissibility, provided that the strain is dominating during the analysis period, and it also correlates with exogenous signals such as mobility indicators and mask wearing. These effects are superimposed in possibly non-linear ways and this approach makes it difficult to deconvolute them; however this can be mitigated by incorporating effects (such as mobility) to the model and appropriately scaling apparent transmissibility. The benefit of this approach is ability to continuously update the projections without explicitly introducing new data or model changes, especially when the population behavior changes rapidly, and it compensates for minor changes in the disease characteristics. We chose this approach to provide continous operational support.

### 4.3. Scenario flexibility

The presented model allows modification of several parameters that can be used to construct different scenarios (Table 3). The primary elements (disease parameters, vaccine effectiveness, variant transmissibility, and immune escape changes) can be included directly by parameterizing a model. The secondary elements can by modeled by proxy through modulating the effective transmissibility β(t) that can be modified either during the fitting process or at the projection stage. As outlined above, the fitted transmissibility correlates with the different phenomena and can be used as a proxy parameter. We have used a modified β(t) to model changes in the NPI (affecting, for example, mobility, mask wearing, etc.), strain transmissibility (in early models we used an increase in transmissibility to model emerging variants), and seasonal changes. The difficulty of implementing these types of projections lies in the fact that the relationships between the apparent transmissibility that our model is using and the modeled phenomena is hard to calculate from the first principles; therefore, we usually depend on the data-driven approximations and model the changes based on changes in past fitted β(t) and applying linear changes to β(t) between predetermined setpoints (see fall/winter seasonal forcing in Fig. 3). The notable exception is the variant transmissibility, where transmissibility advantage or increases in $R_{t}$ can be directly applied to β(t) modulated by the strain prevalence (either reported or modeled using the logistic growth function).

### 4.4. Other applications

As mentioned earlier, beyond the COVID-19 Scenario Modeling Hub, this model framework was also adapted for Virginia Department of Health using county-level and health district-level datasets specific for Virginia. Compared to SMH, these scenarios were rerun on a weekly/biweekly cadence and scenario definitions were updated frequently to reflect the changing COVID-19 landscape and policymaker needs. This model was also used for projections for US Department of Defense at the county level for the US.

When the pandemic began in early 2020, health officials were concerned that an influx of infected patients would overtax local hospital systems; however, anticipating where hospitals were likely to become overextended was a challenge. The COVID-19 Medical Resource Demand Dashboard (for US and Virginia) was an interactive web application that used UVA-adaptive weekly hospitalization projections along with regional staffed bed counts to show where hospitals were projected to approach or exceed capacity in the coming weeks based on underlying scenario assumptions. Users could view projections on a map view where they could focus on regions of interest, and they could also assess projected changes over time on time series graphs; they could toggle between different scenarios, hospital capacities, or durations of stay to assess how changing COVID-19 parameters could impact predictions. The dashboard helped Virginia policymakers make decisions regarding allocation of medical resources and personnel from April 2020 until April 2023.

Additionally, UVA-adaptive projections for the US states for the baseline (status quo) scenario were processed into 4-week ahead forecasts and fed to the statistical ensemble submitted to the CDC COVID-19 Forecast Hub (Adiga et al., 2021).

## Supplementary Material

1

## Figures and Tables

**Table 1:** Scenario-specific implementations for SMH rounds.

Round	Description
11, 12	Two-strain, three-dose vaccination model. **Axis 1: Omicron immune escape vs transmissibility** Modification of immune-escape matrix and strain specific scaling of *β*(*t*) according to modeled pre-Omicron vs Omicron proportions **Axis 2: Omicron severity** Result postprocessing, modification of the projected severity according to variant proportions and ratio of infections coming from individuals with partial protections
13	Three-strain (pre-Omicron, Omicron, new variant), three-dose vaccination model. Seasonal effects included as relative increase in projected *β*(*t*) as observed in past seasons. **Axis 1: Emergence of a new variant** Modification of immune-escape matrix. Forced modeled pre-Omicron/Omicron proportions, new variant simulated unconstrained after seeding proportionally to state population **Axis 2: Waning of protection** Prescribed waning and partial protection parameters included directly in the model
14	Four-strain (pre-Omicron, {BA.1, BA.2, BA.2.12}, {BA.4, BA.5}, new variant), four-dose vaccination model. Seasonal effects included as above. **Axis 1: Emergence of a new variant** as above **Axis 2: Booster strategies** schedules modeled externally
15	Six-strain (pre-Omicron, BA.1, BA.2, BA.2.12, {BA.4, BA.5}, new variant), four-dose vaccination model (first, second, boosters, reformulated booster). Seasonal effects included as above. **Axis 1: Emergence of a new variant** as above **Axis 2: Reformulated booster** schedules modeled externally
16	Seven-strain (pre-Omicron, BA.1, BA.2, BA.2.12, {BA.4, BA.5}, {BQ.1, BA.2.75.2} (level 5), BQ.1.1 (level 6/7)), four-dose vaccination model (first, second, boosters, reformulated booster). Seasonal effects included as above. The level 5 or level 6/7 strains were disabled for respective scenarios by setting their transmissibility to 0 **Axis 1: Emergence of a new swarms of various levels** Modification of immune-escape matrix. Using proportions as reported by CDC at HHS level. Seeding level 5 or level 6/7 strains based on reported proportions, then simulating unconstrained **Axis 2: Reformulated booster** schedules modeled externally

**Table 2:** Transmissibility advantage inferred from the fitted model.

Variant name	Max proportion (per state)	Inferred advantage wrt. ancestral	Reported advantage
Alpha	1	58% (42%−74%)	56%−63% (for USA) (Davies et al., 2021)
Beta	0.12	5% (−764%−774%)	15%−18% wrt. Alpha (Roquebert et al., 2021) (~82%−88% wrt. ancestral)
Gamma	0.32	−12% (−89%−65%)	70%−140% (Faria et al., 2021)
Delta	1	153% (141%−164%)	63%−167% wrt. Alpha (Earnest et al., 2022), (~95%−320% wrt. ancestral)
Epsilon	0.70	88% (41%−135%)	18.6%−24% (Deng et al., 2021)
Iota	0.39	121% (64%−179%)	35% (Annavajhala et al., 2021)
Mu	0.30	372% (195%−549%)	250% (Jimenez-Silva et al., 2022)
Omicron	1	149% (138%−159%)	

**Table 3:** Types of interventions/scenarios.

Intervention	Implementation	Limitations
NPI/mobility/behavior	proxy through *β*(*t*) change	Needs calibrated reference values or heuristics
seasonal effects	proxy through *β*(*t*) change	Needs calibrated reference values or heuristics
disease parameters (waning)	specified directly	
variants (transmissibility)	specified directly through *β*(*t*) change	
variants (immune escape)	specified directly	
vaccine efficacy/protection	specified directly	
vaccine coverage (uptake/hesitancy/rollout)	specified directly	No age-stratification
severity	specified directly through outcome postprocessing	
age specific vaccinations	specified at population level, recovered by postprocessing	under/overestimation of effects depending on current age-specific bias in the population
