# Supplementary material for: Data-driven mechanistic framework with stratified immunity and effective transmissibility for COVID-19 scenario projections
Source: Epidemics. Author manuscript; Available in PMC 2024 Jun 26. (PMC11205267; doi:10.1016/j.epidem.2024.100761)
Supplement: 1 [file NIHMS2002863-supplement-1.pdf]

6. Supplementary information

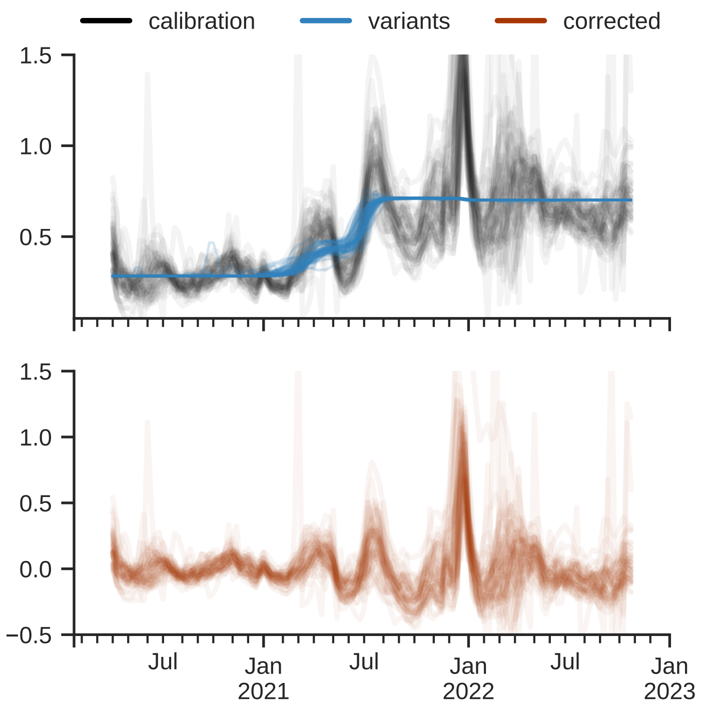

Fig. S1: The $\tilde{\beta}(t)$ for all US states, with fitted contribution from SARS-CoV-2 variants. Each line corresponds to a single US state.

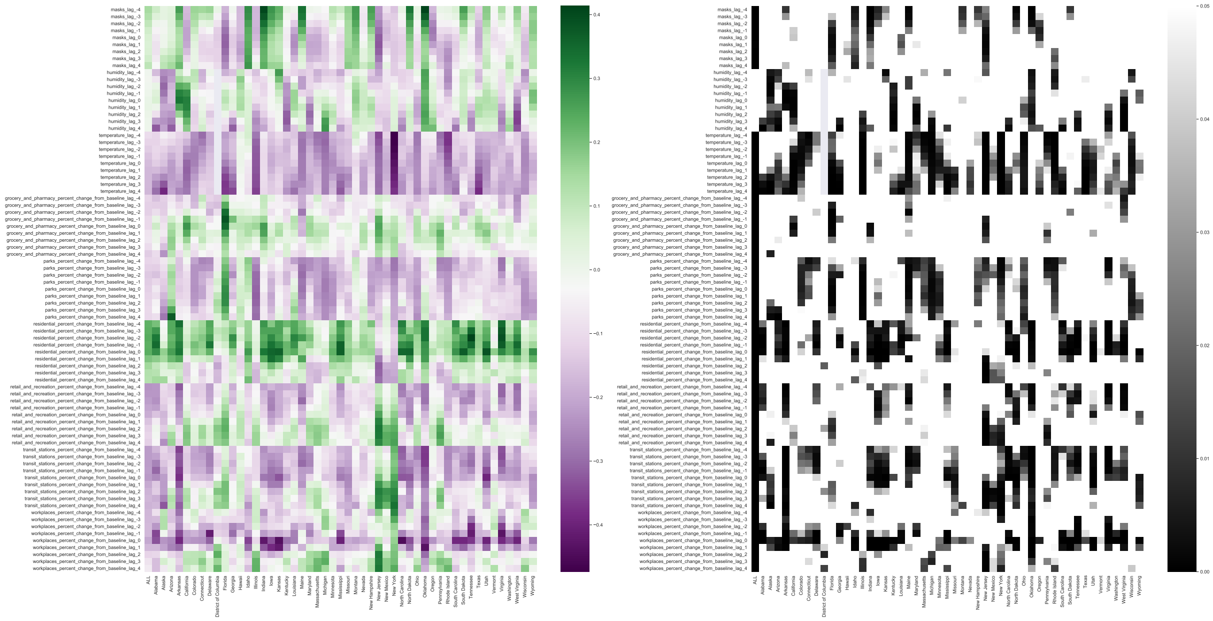

Fig. S2: Pearson Correlation Coefficient (left) and p-value (right) between $\hat{\epsilon}(t)$ and selected metrics and their lagged time series.

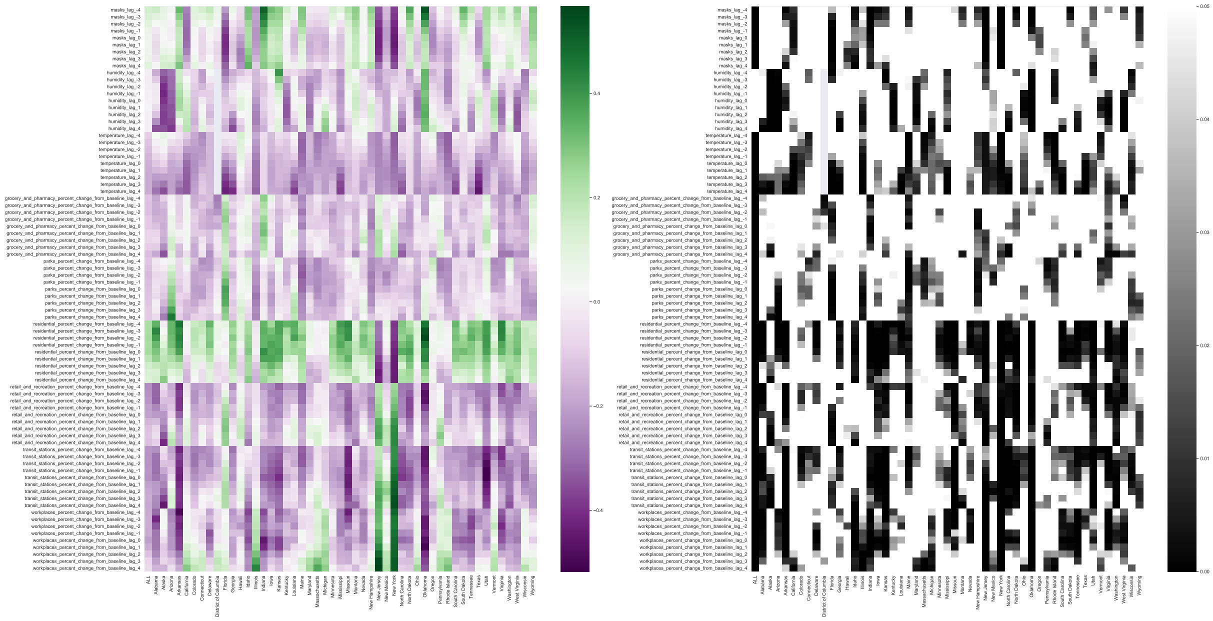

Fig. S3: Spearman Correlation Coefficient (left) and p-value (right) between $\hat{\epsilon}(t)$ and selected metrics and their lagged time series.

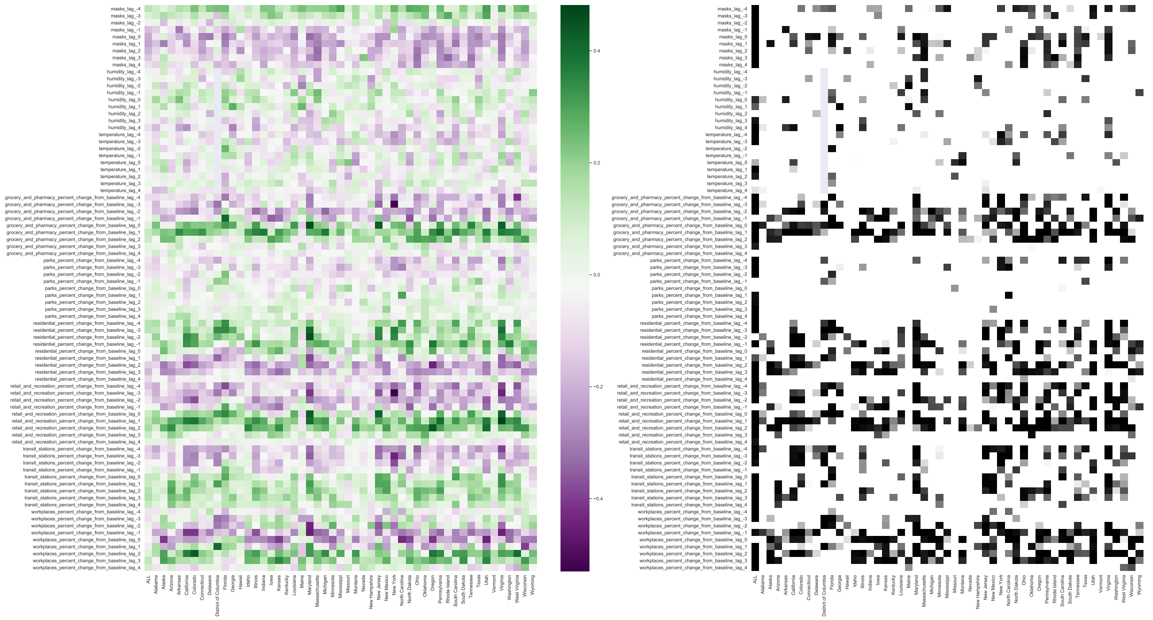

Fig. S4: Pearson Correlation Coefficient (left) and p-value (right) between $\Delta\hat{\epsilon}(t)$ and selected metrics and their lagged time series (differenced).

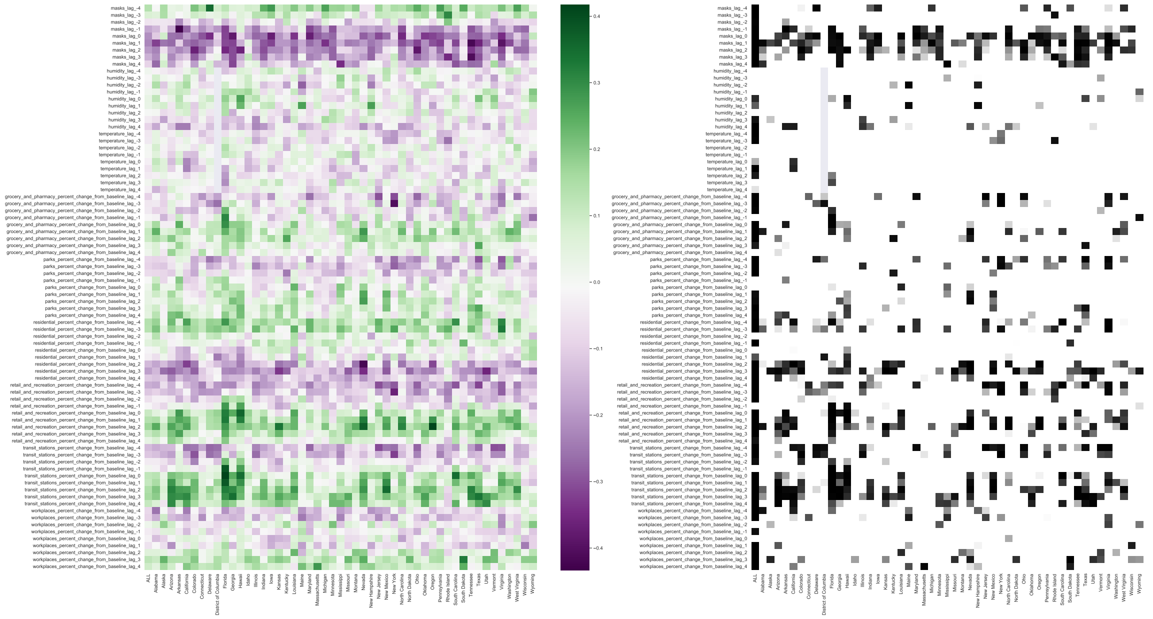

Fig. S5: Spearman Correlation Coefficient (left) and p-value (right) between $\Delta\hat{\epsilon}(t)$ and selected metrics and their lagged time series, (differenced).

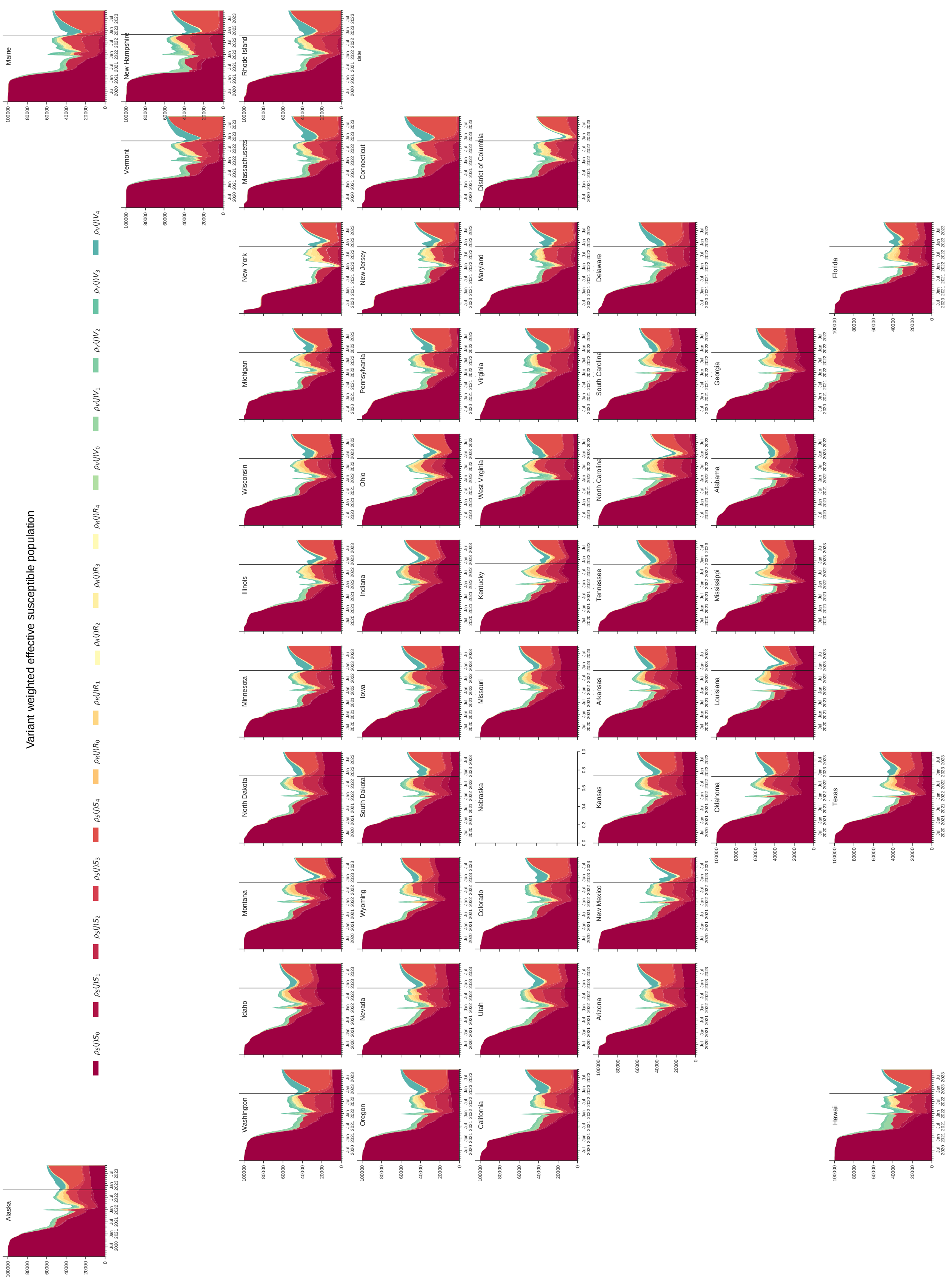

Fig. S6: Effective susceptible populations (per 100,000) for US states

symbol/s	description	SMH values
$\mathbf{M}$	set of strains	[0, 7] (naive, pre-Omicron variants, BA.1, BA.2, BA.2.12.1, {BA.4/5, BA.2.75}, {BQ.1, BA.2.75.2}, BQ.1.1)
$\mathbf{G}$	set of vaccination doses	$\{0, 1, 2, 3, 4, 5\}$ (no dose, first dose, second dose, booster, second booster, bivalent booster)
m, n	single strain; $m, n \in \mathbf{M}$	
i, j, k	vaccination dose; $i, j, k \in \mathbf{G}$	
$\{S, E, I, R, V\}$	compartmental model states (any strain/vaccination dose); susceptible, exposed, infected, recovered, vaccinated; e.g. $S = \sum_{i \in \mathbf{G}, m \in \mathbf{M}} S_i^m$	
$\{S, E, I, R, V\}_i^m$	compartment after dose i and infection with strain m	
$\{S, E, I, R, V\}^m$	combined compartments after infection with strain m regardless of vaccination status; e.g. $S^m = \sum_{i \in \mathbf{G}} S_i^m$	
$\{S, E, I, R, V\}_i$	combined compartments after dose i regardless of infecting strain; e.g. $S_i = \sum_{m \in \mathbf{M}} S_i^m$	
$\{\dot{S}, \dot{E}, \dot{I}, \dot{R}, \dot{V}\}_i^m$	change of the specified compartment during next time step	
$x^m(t, A, B, ...)$	new exposures at a time t to to strain m, coming from compartments $A, B, ...$; $x^m(t, A, B) = x^m(t, A) + x^m(t, B)$	
$x(t, A, B, ...)$	new exposures at a time t to all strains combined, $x(t, \cdot) = \sum_m x^m(t, \cdot)$	
$v_i(t, A, B, ...)$	new vaccination doses i applied to population in compartments A, B, ... $v_i(t, A, B) = v_i(t, A) + v_i(t, B)$	based on CDC data [34] and acceptance surveys [20]

Table S1: Model parameters. Underlined parameters were included in the factorial design for the SMH projections

$\beta(t), \tilde{\beta}(t), \hat{\epsilon}(t)$	apparent transmissiblity; calibrated, median from all particles, residual after including strain contributions	calibrated $\beta(t)$
$\underline{1/\alpha}$	mean duration of incubation	$\{5, 6, 7, 8, 9\}$ days based on 6 days mean from early CDC estimates [35], reduced by 30% for Omicron
$\underline{1/\gamma}$	mean duration of infectiousness	$\{2, 3, 4, 5, 6, 7\}$ days [36], reduced by 30% for Omicron
μ_r, μ_v	rates of waning from natural infection and vaccination	1/(6 months) pre-Omicron, 1/(4 months) Omicron variants, or as prescribed by SMH scenarios
$\underline{\delta_c}$	time difference between the day of exposure to the day of case report	[4, 12] days, based on early CDC estimates
δ_h, δ_d	time difference between the day of case report to the day of hospitalization/death report	calibrated per region at the date of projection using last 2 weeks of available data
$\rho^S_{mni}, \rho^R_{mni}, \rho^V_{mni}$	partial susceptibilities (after waning, recovery, or vaccination) against strain m given prior infection with strain n and vaccination dose i.	based on SMH scenario specification for vaccine efficacy and cross-immunity matrix
ρ^H, ρ^D	case to hospitalization and case to death rates	calculated per region at the date of projection using last 2 weeks of available data (cases – JHU [37], deaths – JHU [37], hospitalizations – HealthData.gov [38])
$\underline{\eta(t), \eta`(t)}$	case ascertainment rate, home-test adjusted case ascertainment rate	+/- 20% from values calculated using CDC seroprevalence data [39] and home test surveys [16]
$P, p(t), C(t)$	population, fraction of people with past SARS-CoV-2 infection, cumulative cases; region specific	CDC seroprevalence data [39]
$c(t), h(t), d(t)$	projected cases, hospitalizations and deaths	

Table S2: Model parameters (cont.). Underlined parameters were included in the factorial design for the SMH projections

7. Example equations for two strain, single vaccination model

The two strain $m \in \{0, 1, 2\}$, single vaccination $i \in \{0, 1\}$ model has the following compartments:

S_0^0 – fully susceptible population (no immunity)
S_1^0 – waned after vaccination (partial protection)
V_1^0 – vaccinated

S_0^1 – waned after infection with strain 1 (partial protection)
E_0^1 – exposed with strain 1
I_0^1 – infectious with strain 1
R_0^1 – recovered from strain 1 infection (full protection, leaky)

S_0^2 – waned after infection with strain 2 (partial protection)
E_0^2 – exposed with strain 2
I_0^2 – infectious with strain 2
R_0^2 – recovered from strain 2 infection (full protection, leaky)

S_1^1 – waned after infection with strain 1 and vaccination (partial protection)
E_1^1 – exposed with strain 1 and after vaccination
I_1^1 – infectious with strain 1 and after vaccination
R_1^1 – recovered from strain 1 infection and after vaccination (full protection
V_1^1 – vaccinated and after infection with strain 1 (full protection, leaky)

S_1^2 – waned after infection with strain 2 and vaccination (partial protection)
E_1^2 – exposed with strain 2 and after vaccination
I_1^2 – infectious with strain 2 and after vaccination
R_1^2 – recovered from strain 2 infection and after vaccination (full protection, leaky
V_1^2 – vaccinated and after infection with strain 2 (full protection, leaky)

$$\dot{S}_0^0 = -x^1(t, S_0^0) - x^2(t, S_0^0) - v_1(t, S_0^0)$$

$$\dot{S}_1^0 = -x^1(t, S_1^0) - x^2(t, S_1^0) + \mu_v V_1^0$$
$$\dot{V}_1^0 = v_1(t, S_0^0) - \mu_v V_1^0 - x^1(t, V_1^0) - -x^2(t, V_1^0)$$

$$\dot{S}_0^1 = -x^1(t, S_0^1) - x^2(t, S_0^1) + \mu_r R_0^1 - v_1(t, S_0^1)$$
$$\dot{E}_0^1 = x^1(t, S_0^0) + x^1(t, S_0^1, R_0^1) - \alpha E_0^1$$
$$\dot{I}_0^1 = \alpha E_0^1 - \gamma I_0^1$$
$$\dot{R}_0^1 = \gamma I_0^1 - \mu_r R_0^1 - x^1(t, R_0^1) - x^2(t, R_0^1) - v_1(t, R_0^1)$$

$$\dot{S}_0^2 = -x^2(t, S_0^2) + \mu_r R_0^2 - v_1(t, S_0^2)$$
$$\dot{E}_0^2 = x^2(t, S_0^0) + x^2(t, S_0^1, R_0^1) + x^2(t, S_0^2, R_0^2) - \alpha E_0^2$$
$$\dot{I}_0^2 = \alpha E_0^2 - \gamma I_0^2$$
$$\dot{R}_0^2 = \gamma I_0^2 - \mu_r R_0^2 - x^2(t, R_0^2) - v_1(t, R_0^2)$$

$$\dot{S}_1^1 = -x^1(t, S_1^1) - x^2(t, S_1^1) + \mu_r R_1^1 + \mu_v V_1^1$$
$$\dot{E}_1^1 = x^1(t, S_1^0, V_1^0) + x^1(t, S_1^1, R_1^1, V_1^1) - \alpha E_1^1$$
$$\dot{I}_1^1 = \alpha E_1^1 - \gamma I_1^1$$
$$\dot{R}_1^1 = \gamma I_1^1 - \mu_r R_1^1 - x^1(t, R_1^1) - x^2(t, R_1^1)$$
$$\dot{V}_1^1 = v_1(t, S_0^1, R_0^1) - \mu_v V_1^1 - x_1(t, V_1^1) - x_2(t, V_1^1)$$

$$\dot{S}_1^2 = -x^2(t, S_1^2) + \mu_r R_1^2 + \mu_v V_1^2$$
$$\dot{E}_1^2 = x^2(t, S_1^0, V_1^0) + x^2(t, S_1^1, R_1^1, V_1^1) + x^2(t, S_1^2, R_1^2, V_1^2) - \alpha E_1^2$$
$$\dot{I}_1^2 = \alpha E_1^2 - \gamma I_1^2$$
$$\dot{R}_1^2 = \gamma I_1^2 - \mu_r R_1^2 - x^2(t, R_1^2)$$
$$\dot{V}_1^2 = v_1(t, S_0^2, R_0^2) - \mu_v V_1^2 - x_2(t, V_1^2)$$

8. Derivation of cross-immunity matrix from NT50 values.

Let's define the immune escape at the molecular level as the relative difference between bound fractions f^b of two antigens reference ref and escaping esc bound to an antibody Ab, with dissociation constants $K_d^{ref} < K_d^{esc}$:

$$IE = (f_b^{ref} - f_b^{esc})/f_b^{ref}$$

Given the definition of the dissociation constant:

$$K_d = \frac{[Ag][Ab]}{[AgAb]}$$

where $[Ag], [Ab], [AgAb]$ are the concentrations of the free antigen, free antibody and antigen-antibody complex the fraction of antigen bound to antibody is defined as:

$$f_b = \frac{[AgAb]}{[Ag] + [AgAb]} = \frac{[Ab]}{[Ab] + K_d}$$

from there the IE can be expressed in terms of K_d as:

$$IE = \frac{\frac{[Ab]^{ref}}{[Ab]^{ref}+K_d^{ref}} - \frac{[Ab]^{esc}}{[Ab]^{esc}+K_d^{esc}}}{\frac{[Ab]^{ref}}{[Ab]^{ref}+K_d^{ref}}}$$

because under pseudovirus neutralization titer assay conditions the total concentration of antigen $[Ag^{tot}]$ (in $< 10^{-12}$M range [40, 41, 42]) is significantly lower than concentration of its neutralizing antibody $[Ab^{tot}]$ (in the 10^{-6}M – 10^{-9}M range, [43]) then $[Ab] \approx [Ab_{tot}]$ regardless of antigen concentration, therefore:

$$IE = \frac{\frac{[Ab^{tot}]}{[Ab^{tot}]+K_d^{ref}} - \frac{[Ab^{tot}]}{[Ab^{tot}]+K_d^{esc}}}{\frac{[Ab^{tot}]}{[Ab^{tot}]+K_d^{ref}}} = 1 - \frac{K_d^{ref} + [Ab^{tot}]}{K_d^{esc} + [Ab^{tot}]}$$

The apparent K_d of the serum is not known, however the relation of the K_d^{ref} and K_d^{esc} can be expressed relative to observed dilutions with 50% inhibitory effect (NT50) as:

$$K_d^{esc} = wK_d^{ref}, w = \frac{NT50_{ref}}{NT50_{esc}}$$

i.e. the twofold reduction of the NT50 corresponds to twofold increase in K_d. Finally, at the conditions when NT50 is determined (half of the viral particles are neutralized, i.e. half of the antigen is bound to antibody) $[Ag^{NT50}] = [AgAb^{NT50}]$ and the total antibody concentration $[Ab^{tot}] \approx [Ab^{NT50}]$. Therefore:

$$K_d = \frac{[AgAb^{NT50}][Ab^{NT50}]}{[AgAb^{NT50}]} = [Ab^{NT50}] \approx [Ab^{tot}]$$

$$IE = 1 - \frac{K_d^{ref} + [Ab^{tot}]}{eK_d^{ref} + [Ab^{tot}]} = 1 - \frac{K_d^{ref} + K_d^{ref}}{wK_d^{ref} + K_d^{ref}} = 1 - \frac{2}{w+1} = \frac{w-1}{w+1}$$
